# Development and On-Field Testing of Low-Cost Portable System for Monitoring PM2.5 Concentrations

**DOI:** 10.3390/s18041056

**Published:** 2018-04-01

**Authors:** Konstantinos N. Genikomsakis, Nikolaos-Fivos Galatoulas, Panagiotis I. Dallas, Luis Miguel Candanedo Ibarra, Dimitris Margaritis, Christos S. Ioakimidis

**Affiliations:** 1 European Research Area Chair (*Holder) ‘Net-Zero Energy Efficiency on City Districts, NZED’ Unit, Research Institute for Energy, University of Mons, Rue de l’Epargne, 56, 7000 Mons, Belgium; konstantinos.genikomsakis@umons.ac.be (K.N.G.); nikolaos-foivos.galatoulas@umons.ac.be (N.-F.G.); luiscandanedoi@gmail.com (L.M.C.I.); 2 Wireless Network Systems Division, INTRACOM Telecom S.A., 19.7 km Markopoulo Ave., Peania, 19002 Athens, Greece; pdal@intracom.gr; 3 Centre for Research and Technology Hellas (CERTH), Hellenic Institute of Transport (HIT), 6th km Charilaou-Thermi, 57001 Thermi, Thessaloniki, Greece; dmarg@certh.gr

**Keywords:** air pollution, calibration, environmental monitoring, mobile applications, particulate matter, sensor systems, system testing

## Abstract

Recent developments in the field of low-cost sensors enable the design and implementation of compact, inexpensive and portable sensing units for air pollution monitoring with fine-detailed spatial and temporal resolution, in order to support applications of wider interest in the area of intelligent transportation systems (ITS). In this context, the present work advances the concept of developing a low-cost portable air pollution monitoring system (APMS) for measuring the concentrations of particulate matter (PM), in particular fine particles with a diameter of 2.5 μm or less (PM2.5). Specifically, this paper presents the on-field testing of the proposed low-cost APMS implementation using roadside measurements from a mobile laboratory equipped with a calibrated instrument as the basis of comparison and showcases its accuracy on characterizing the PM2.5 concentrations on 1 min resolution in an on-road trial. Moreover, it demonstrates the intended application of collecting fine-grained spatio-temporal PM2.5 profiles by mounting the developed APMS on an electric bike as a case study in the city of Mons, Belgium.

## 1. Introduction

Road transportation is known to be a major source of air pollution with particularly adverse effects on the natural environment and quality of human life, calling for drastic actions to reduce the levels of harmful gases and particulate matter (PM), mainly in urban areas. Over the years, there have been significant efforts to develop a variety of applications relevant to intelligent transportation systems (ITS), not only for improving traffic flow or reducing emissions and energy consumption from transportation related activities [1] but also for monitoring air pollution [2]. Existing solutions for air pollution monitoring include networks of static measurement stations, characterized by high accuracy and reliability as well as ability to measure a wide range of air pollutants. However, the installation of these systems faces severe limitations due to high acquisition and maintenance costs [3]. From the portability point of view, vehicles equipped with on-board costly measurement devices can be used as mobile laboratories for air pollution monitoring with high spatial and temporal resolution [4].

It becomes clear that there is a challenging field of research with respect to the development of cost-effective portable devices that can be mounted on vehicles in order to support applications of wider interest in the area of ITS, for instance, portable sensing units on public transportation vehicles for real-time display of pollutants levels [5] and generation of high-resolution urban air pollution maps [3], on conventional bikes for mobile air quality monitoring [6,7] or on electric bikes for capturing fine-grained environmental information and planning healthier routes [8,9]. Combining the available information and communications technologies (ICT) with the recent developments in the market of low-cost sensors enables not only the design and implementation of compact, inexpensive and portable sensing units for creating profiles of aerosol concentrations with fine-detailed spatial and temporal resolution but also the deployment of networks of such devices as mobile agents operating concurrently under a big data analysis scheme in order to develop applications and provide add-on services in the context of ITS. The authors in [10] emphasize on the need to quantify the performance of these low-cost sensors under real-world conditions. To that end, field experiments are employed in [11] to examine the consistency, stability and durability of the sensing units, using hourly data for PM concentrations.

In this context, the focus of the present work is on low-cost sensors for implementing a portable unit to measure the concentration of PMs, in particular fine particles with a diameter of 2.5 μm or less (PM2.5), the exposure to which is known to cause acute and chronic effects on human health, including premature mortality due to cardiopulmonary diseases and lung cancer [12]. Specifically, this paper introduces the key components of a portable air pollution monitoring system (APMS) based on low-cost sensors, describes the on-field testing of the proposed sensing unit using roadside measurements from a mobile laboratory equipped with a calibrated instrument as the basis of comparison and showcases its accuracy on characterizing the PM2.5 concentrations on 1 min resolution in an on-road experiment. Moreover, it presents a case study to demonstrate the intended application by mounting the proposed low-cost APMS on an electric bike (e-bike) in order to collect data of PM2.5 concentrations in the city of Mons, Belgium and compare them with the hourly measurements available from the local monitoring station. At this point, it is noted that the purpose of this work is neither to detect the source of the pollutants nor to chemically characterize them but rather to propose a low-cost mobile measurement system to map ambient PM concentration levels stemming from diverse activities in urban environments.

Specifically, this work examines the potential of a portable APMS with integrated low-cost sensor technologies to perform reliable measurements of PM2.5 concentrations in the ambient air after on-field calibration with a certified optical particle counter instrument. Mobile monitoring of air pollution with an electric vehicle is demonstrated for determining and comparing emission factors from vehicles under various conditions in [13], assessing on road near-source air quality gradients in [14] and characterizing exposure zones in [15]. Taking into account the above, the main contributions of this work include the following: (i) the implementation of APMS is based on a PM sensor that has not been reported in the literature before; (ii) roadside and on-road measurements were carried out to experimentally test and validate the proposed APMS using a mobile air pollution laboratory housed in a van as a calibrated comparison system; (iii) regression models were derived from post-processing of the sensor readings in order to assess ambient PM2.5 concentrations in a geo-referenced mobile test; and (iv) an e-bike was employed as a case study to demonstrate the intended application, along with the capability of collecting fine-grained spatio-temporal contamination profiles, in sharp contrast to the hourly averages of PM2.5 concentrations from fixed monitoring stations.

The rest of the paper is structured as follows: Section 2 provides an overview of the relevant work in the fields of mobile air pollution monitoring and roadside measurements. Section 3 presents the key components of the proposed low-cost APMS and the mobile laboratory, as well as describes the experimental campaign. Section 4 focuses on the calibration of the APMS based on stationary measurements, while Section 5 assesses the validity of the developed models under a mobile run test. Section 6 discusses the results obtained in an urban case study, where the APMS was mounted on an e-bike. Last, Section 7 draws the main conclusions of the paper.

## 2. Related Work

In recent years, there has been growing research interest in developing low-cost portable APMSs that can provide fine-grained data related to traffic induced emissions in urban areas, employing the latest advancements in big data analysis and sensor node infrastructures communicating with wireless sensor network (WSN) technology [16]. In an attempt to introduce inexpensive air mapping solutions, mobile measurement systems are designed to function in an interoperable multi-vehicular way and tested in applications ranging from pedestrian wearables, bicycles, public transportation, to automobiles [17]. On-road trials with experimental instrumentation have produced benchmark results on the real-time emissions footprint of vehicles [18]. Until now similar methodologies have been applied for a variety of objectives, such as assessment of personal exposure by providing equipment to the study object [19], mapping spatial variation in air pollution [20], or to develop and validate air quality models [21]. Other studies address the potential of using mobile measurements to construct air pollution maps at a high spatial resolution [22]. Mobile monitoring techniques also receive an increasing attention for participatory sensing and crowdsourcing methods [23,24], hence setting the field for urban citizen science to investigate the correlation between air quality and public health. In this context, community-based sensing projects proposed novel low-cost APMS instrument for large-scale and mobile deployment [25].

Roadside measurements provide the instantaneous ratios of pollutant concentrations from vehicles passing by the roadway where the station is installed. Mobile-sensing enables monitoring of emissions linked to a large number of vehicles with a tuneable spatio-temporal resolution to match emission levels with specific vehicles [26]. Currently, this sensing approximation provides a roughly estimated value of PM concentrations, mainly sourcing from fuel combustion, exhausts, tires and secondary particles formed in the atmosphere at a specific location under specific operating conditions and it relies on the evolution of the current inexpensive sensing components in order to produce more trustworthy quantitative models of heavy traffic [27]. Thus, it is a challenging task to develop emission factor models that better capture the PM footprint from various sources, depending solely on inexpensive mobile-sensing instruments. Furthermore, designing mobile-sensing systems needs to carefully deal with unstable weather and background pollution levels that hinder the evaluation of the actual vehicle emissions. In particular, the performance of three inexpensive sensing devices when measuring engine exhaust PM emissions are evaluated in [28].

## 3. Description of Low-Cost APMS and Experimental Setting

This section describes in detail the key components of the proposed APMS implementation using low-cost sensors and provides an overview of the mobile laboratory that was employed as a comparison measurement system for the on-field evaluation.

### 3.1. Design Specifications of Low-Cost APMS

**Figure 1:**
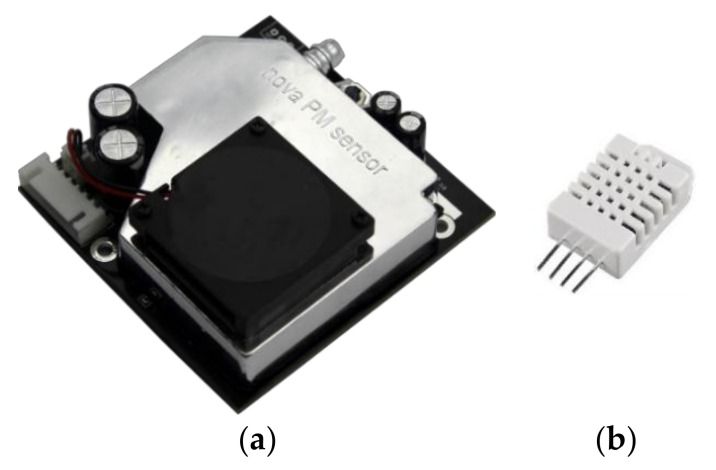
Low-cost sensors: (**a**) SDS011 for measuring concentrations of PM2.5 and PM10; (**b**) DHT22 for measuring temperature and relative humidity.

**Figure 2:**
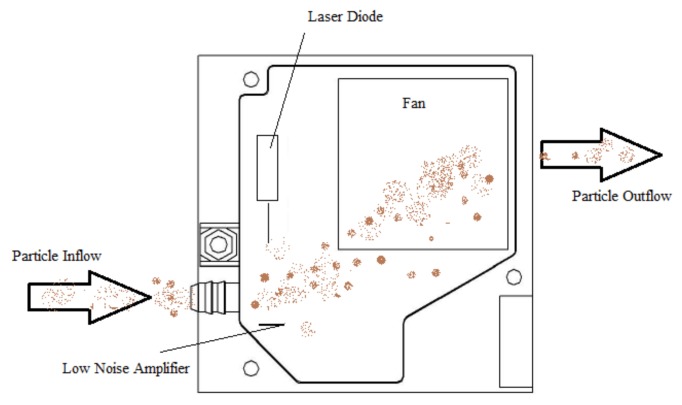
Layout of SDS011 sensor for measuring particulate matter (PM) concentrations. Particles flow through the sampling inlet and enter the sensing chamber, where the incident laser beam is scattered depending on their size. A low noise amplifier operates as a transducer, by transforming the collected light to various signal intensities.

The proposed low-cost APMS implementation is aimed to function as a portable, compact and adjustable data collecting solution. It consists of a single board microcontroller equipped with a data-logging extension, a GPS module and an off-the-shelf optical PM sensor, namely Laser PM2.5 Sensor SDS011 by Nova Fitness [29] shown in Figure 1a. The sensor is designed with a built-in fan to ensure sample air circulation to a chamber with a laser diode, where the size and amount of PM is determined. The scattered light is transformed into electrical signals and with further analysis on the signal waveform the digital output of the component is the particle concentration of PM2.5 and PM10 (from counts of particles with diameters from 0.3 to 10 μm). The working principle is based on light scattering, where a light source illuminates the particles and the scattered light is transformed into a signal by a photodetector, the amplitude of which depends on the light wavelength, scattering angle, particle size and relative index of refraction between medium and particle. The sensor under study uses sampling with 0–5 V pulses of 1004 ms in length, allowing measurement intervals of roughly 1 s. In detail, a laser diode generates a single frequency beam, with typical wavelength in similar components ranging from 870 to 980 nm (infrared spectrum) as reported in [30]. In contrast to the other commercially available low-cost PM sensors described in [30] that use thermal resistors to generate heat and thus employ natural convection for introducing the particles in the light scattering region, the apparatus under study achieves that with an embedded fan that generates a negative pressure in order to conduct the particle flow through the specified path (Figure 2). The SDS011 sensor measures PM2.5 and PM10 within the range 0.0 to 999.9 µg/m^3^ and it has a maximum relative error of ±15% at 25 °C and 50% relative humidity, according to the manufacturer. At this point, it is noted that the specific sensor yielded some errors in the measurements (PM2.5 concentration = 999.9 µg/m^3^) taken in the frame of this work, thus these outlier readings were filtered-out from the time series presented in the next sections of the paper.

Thereafter, the microcontroller was programmed to record the timestamped reading of the PM sensor. The data logger of the low-cost APMS records measurements from the on-board sensors every 4 s. The logger has a real-time clock that is used to add a timestamp to each measurement and saves the information in an individual comma-separated values (csv) file each day. Auxiliary sensor components for temperature and relative humidity were added to the system in order to examine the effects of ambient weather conditions on the overall performance and thereby to exclude any possible alterations on the quality of the collected data. For the purposes of this work, the DHT22 sensor was chosen to measure temperature and relative humidity (Figure 1b), given that it is a manufacturer-calibrated digital sensor that offers an accuracy of ±0.5 °C for temperature and ±2% (maximum ±5%) for relative humidity.

The final assembly along with a powering circuit enabling it to power from a range of storage options was cased in a polypropylene box with dimensions 27 × 11 × 7 cm and weight 150 g. In cost terms, the electronic parts were acquired from online retailers, with a total material cost rising up to 120 €. However, it is important to note that an even more cost-effective variant, which assigns the power and data logging to a portable computer, could be constructed for less than 80 €.

### 3.2. Comparison Measurement System

**Figure 3:**
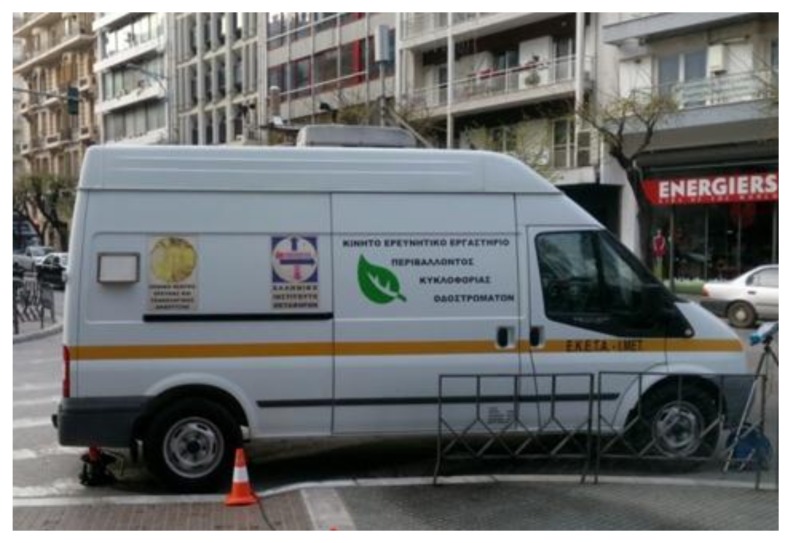
Mobile Lab for Environmental and Traffic Measurements of Centre for Research Technology (CERTH)/Hellenic Institute of Transportation (HIT), equipped with a portable air pollution sensor rack for collecting and analysing environmental pollution and traffic related data.

Field experiments for tuning and calibrating the described prototype were carried out in collaboration with the Hellenic Institute of Transportation (HIT) at the Centre for Research Technology (CERTH) in Thermi-Thessaloniki, Greece. The provided comparison system was the Mobile Lab for Environmental and Traffic Measurements (Figure 3) equipped with a certified PM concentration analyser. The Mobile Lab carries automatic gas emissions analysts, series AP-370 by HORIBA, suitable for the constant air pollution measurement, which are certified according to international standards organizations, that is, US EPA and TUV. They are state-of-the-art technology, controlled by an embedded processor, have an embedded sample pump and keyboard, provide instant values on a digital screen, full diagnostics both on screen and on RS232 port and interact with the proper systems for the sampling, calibration, data recording-processing and communication with the central station.

A portable high quality PM2.5 sensor, namely the Optical Particle Sizer (TSI OPS 3330), was used to compare the measurements from the SDS011 sensor. The former is based on an optical principle of operation with 120° light collection and electronics processing resulting in precise, high quality data. It can detect particles in the size range of 0.3–10 μm in up to 16 channels and on a concentration range from 0 to 3000 particles/cm^3^. A built-in pump in this instrument fixes the flow rate (sample and sheath) to 1 L/min. At this point, it is important to note that, according to the instrument’s certificate of calibration and testing, it has been calibrated using standards whose accuracies are traceable to the United States National Institute of Standards and Technology (NIST) or has been verified with respect to instrumentation whose accuracy is traceable to NIST. Moreover, data for both temperature and humidity parameters were available from the stationed Mobile Lab. The data-sampling rate of TSI OPS 3330 was initially set to 1 sample reading per min for collecting stationary measurements and was later raised to 1 sample reading per 10 s for collecting measurements en route. The data collected from the loggers of the two different devices (i.e., of TSI 3330 OPS and proposed low-cost APMS) were averaged by 1 min to establish a common time basis for comparison purposes.

### 3.3. Experimental Campaign

The experimental campaign took place from 6–8 March 2017 in the city of Thessaloniki, Greece, with the aim to monitor PM emissions, in particular PM2.5, from urban traffic with the Mobile Lab for Environmental and Traffic Measurements used as a comparison system. First, a set of roadside measurements was collected in the area of CERTH premises, close to an intersection with high vehicle-traffic and in the vicinity of agricultural facilities. The objective of this field test was to assess the operability of the proposed low-cost APMS implementation under real-world conditions and calibrate its sensors based on data concurrently measured from the specialized high quality optical particle sizing instrument TSI OPS 3330. The duration of the stationary experiment was 3 days and the collected data were stored in a microSD card on the prototype for post-processing.

**Figure 4:**
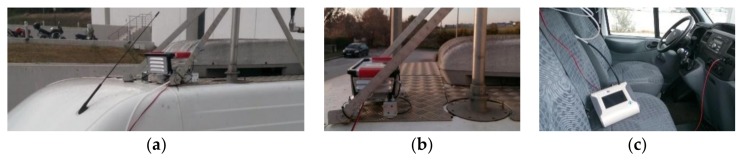
Details of the experimental setup: (**a**) Rooftop installation; (**b**) End of the certified instrument alongside the low-cost air pollution monitoring system (APMS) ensuring common sample air flow; (**c**) Calibrated optical particle counter instrument and power circuit draining voltage from the van battery.

Next, a mobile run test was performed in order to evaluate the accuracy of individual sensors and the performance of the system as a whole in an urban rush-hour emissions measurement scenario. A route with variations in traffic density was designed with the aim to test the PM2.5 sensing validity in areas with previously recorded high level of air contamination concerning these particle size categories (based on past measurements of central stations). The system was mounted on the Mobile Lab rooftop and stabilized at 2.6 m above ground level. The comparison system received sampling air from approximately the same level with the proposed APMS device by properly extending the piping to ensure constant ambient air flow. Following the manufacturer’ instructions, before and after connecting the tubing extension to the inlet of TSI OPS 3300, stationary samples (background measurements) were taken and compared in order to ensure that the particle chamber is not contaminated, thereby the use of the tubing extension did not affect the quality of the measurements. Figure 4 illustrates the mounted low-cost APMS on the rooftop of the Mobile Lab for performing both field experiments.

## 4. Field Calibration with Stationary Measurements

Given that the low-cost APMS records a new measurement every 4 s, some tens of thousands PM sensor readings were made available for calibration purposes in the frame of this work through a stationary experiment. The first set of training data (from roadside measurements) investigated the effect of ambient conditions on the system’s accuracy and compared it with the calibrated instruments of the Mobile Lab. To this end, the collected data from both measurement systems were post-processed with loess smoothers and the resulting plots were examined for linearity. The strengths of correlations and quantitative relationships in the pairwise plots were extracted with the use of the coefficients of determination (R^2^) from fitted ordinary linear regression models. As described in Section 5, the accuracy of the linear calibrations was identified with the computation of well-established error metrics on the test dataset (from the mobile run). In addition, the statistical weights of measured parameters such as temperature and relative humidity on instrument performance were evaluated with sensitivity analysis.

### 4.1. Temperature and Relative Humidity

**Figure 5:**
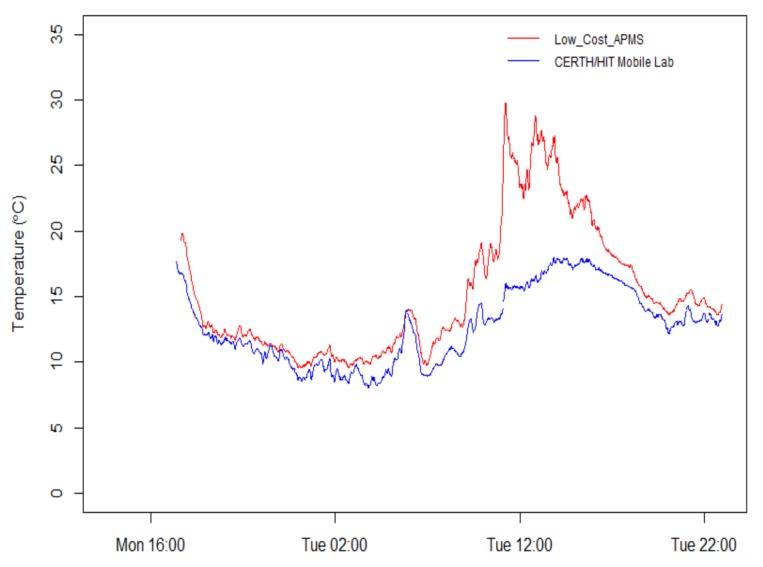
Time series of temperature measurements with 1 min resolution.

Figure 5 presents the measured temperature from the two devices during the roadside stationary run. There is satisfactory agreement between the two instruments with the exception of daylight hours with sunshine. In this case, the electronics housed inside the low-cost APMS provide readings with higher values, most probably due to the solar radiation heating up the enclosure and to the contribution of excess heat from the components. The coefficient of determination between the two time series was calculated and found to be approximately 0.879.

**Figure 6:**
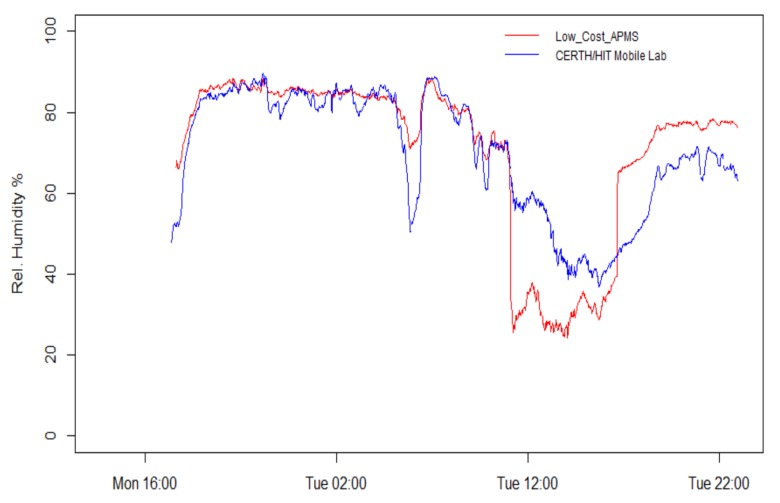
Time series of relative humidity measurements with 1 min resolution.

Relative humidity measured in the box varied from 20% to 90% over the course of the experiment, as detailed in Figure 6. During night hours, the humidity values are almost identical. However, during daytime, the readings are affected by the changes in temperature of the enclosure due to the solar radiation heating up the enclosure. In addition, relative humidity levels from the prototype decrease by excess heat emitted by the housed electronic components. The coefficient of determination between the two time series is high (0.822). As a side observation, the measurements confirm that the recorded humidity levels follow (inversely) the trend of temperature.

### 4.2. PM2.5 Concentrations

**Figure 7:**
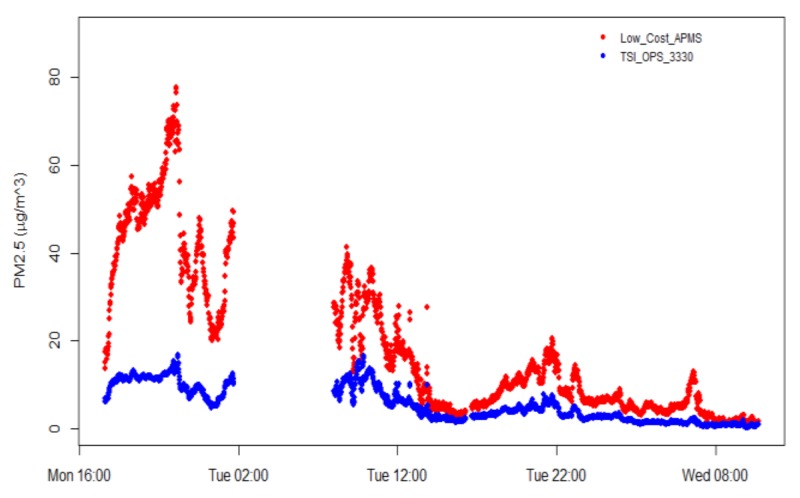
Time series of PM2.5 concentration measurements with 1 min resolution (data collected from March 6 to March 8, 2017).

The time series of PM2.5 concentration data from the instruments deployed outside CERTH/HIT for a period of three days are illustrated in Figure 7. As expected, night-time values were higher than daytime concentrations, directly linked to the descent of the boundary layer. Some recorded data loss during night-time occurred due to the limitations of data storage of the TSI OPS 3330 instrument. At a first glance, there was a significant deviation between the low-cost APMS and the comparison instrument. Although the absolute values are quite different, the correlation between the two PM2.5 readings is high, as the coefficient of determination was 0.932, which is promising for the low-cost sensor, yet suggests that the sensor readings require adjustment in order to improve the accuracy of the low-cost APMS output.

### 4.3. Linear Regression Models

Following the objective to calibrate the low-cost PM2.5 sensor, two linear regression models were fitted in the R programming language [31]. The resulting regression models are given in Equations (1) and (2), with 0.932 and 0.947 coefficients of determination respectively.

(1) $$\mathrm{PM}2.5_{\mathrm{model}1}=1.473073+0.216347\cdot \mathrm{PM}2.5_{\mathrm{sensor}},$$

(2) $$\mathrm{PM}2.5_{\mathrm{model}2}=-8.64763+0.36929\cdot T+0.05850\cdot \mathrm{RH}+0.22606\cdot \mathrm{PM}2.5_{\mathrm{sensor}},$$

where PM2.5_model1_ and PM2.5_model2_ are the adjusted output of the two linear regression models in terms of PM2.5 concentrations, while T, RH and PM2.5 sensor are the sensor readings of temperature, relative humidity and PM2.5 concentrations respectively. 

Regarding the sensitivity analysis, the computed probabilities for model 1 indicated that there is a statistically significant covariance between the PM2.5 readings of TSI OPS 3330 and the PM2.5 concentration measurements from the low-cost sensor (*p* value associated with PM2.5 concentration coefficient <2× 10^–16^). In the case of model 2, the analysis shows that there is also a statistically significant relationship with temperature and relative humidity measurements from the low-cost sensor (*p* values associated to temperature and relative humidity coefficients <2× 10^–16^). Given that the second model presented an increased adjusted R^2^ value (0.947) compared to the first one which assumes a correlation solely between the PM2.5 concentrations of the two instruments, the second model is expected to provide a better prediction.

**Figure 8:**
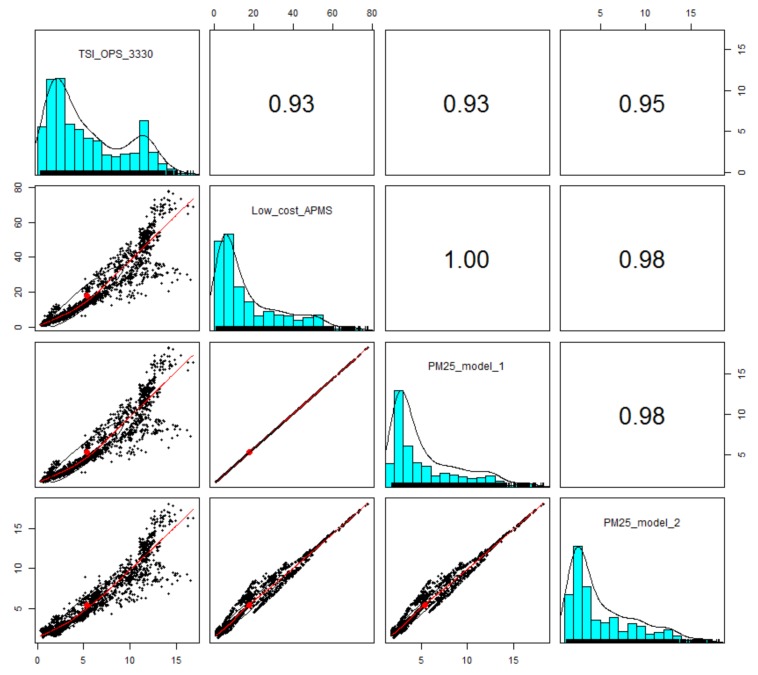
Pairs plot of comparisons between the TSI OPS 3330 measurements, low-cost sensor readings and the output of the derived regression models from the roadside stationary experiment performed from March 6 to March 8, 2017. Upper right section depicts the R^2^ as computed by the Pearson method, while lower-left set of panels present pairwise plots of 1 min averages with loess smoothers applied. It is noted that the plots in the same row have the same scale in the y axis (in μg/m^3^), while the plots in the same column have the same scale in the x axis (in μg/m^3^).

Figure 8 presents a pair-wise plot of the measurements by TSI OPS 3330, readings of the low-cost sensor (SDS011) and the output of the derived regression models in order to verify the linear regression assumption, indicating that the data points are randomly dispersed around the *y* = *x* diagonal and therefore the linear model is appropriate for the PM2.5 range under study (2 to 10 µg/m^3^). As shown in Equation (2), the parameters of temperature and relative humidity are introduced in model 2. Thus, the impact of temperature and relative humidity variations on the raw measurements of the low-cost PM2.5 sensor is shown in the second and fourth panel of the first row of plots in Figure 8, where the value of the coefficient of determination between the raw measurements of the low-cost PM2.5 sensor and those from TSI OPS 3330 increases from 0.93 (model 1) to 0.95 (model 2) when temperature and relative humidity measurements are taken into account in the regression model. As a side observation, it is noted that relevant data from exposure to a highly contaminated environment are needed to improve the accuracy of PM2.5 detection for higher values of concentration.

## 5. Mobile Run Validation

**Figure 9:**
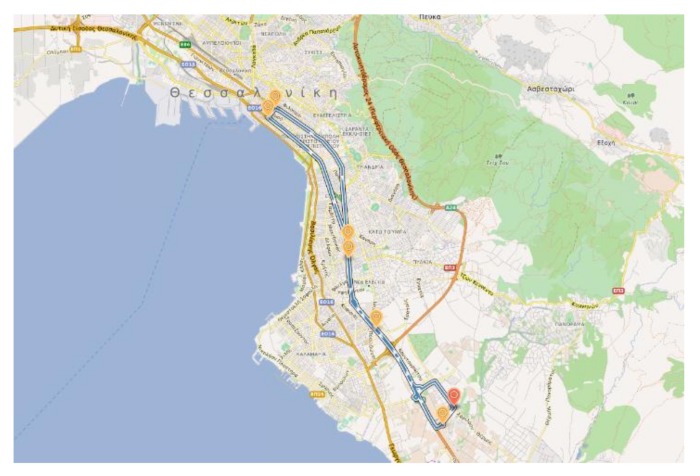
Route followed during the mobile testing in the area of Thessaloniki, Greece on 9 March 2017.

**Figure 10:**
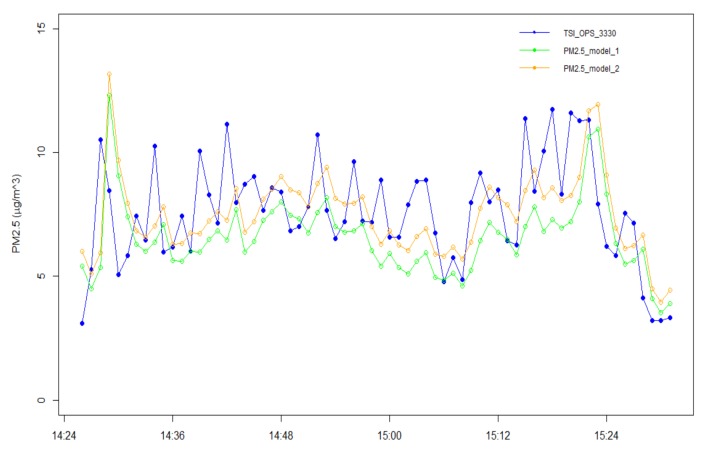
Time series of PM2.5 concentrations with 1 min resolution from the mobile test run in the area of Thessaloniki, Greece on 9 March 2017. The two fitted linear regression models are compared with the readings of the optical particle sizer.

For validation purposes, the resulting linear regression models were tested on data collected during a mobile run in the city of Thessaloniki, Greece, following the route shown in Figure 9. The duration of the route was 1 h and 15 min and high deviations of PM2.5 concentration levels were measured by the TSI OPS 3330 instrument. Figure 10 shows the comparison of the estimated PM2.5 concentrations using both fitted models versus the readings of the certified optical particle sizer. The error metrics, namely root mean squared errors (RMSE), mean absolute error (MAE) and mean absolute percentage error (MAPE), are reported in Table 1, where the second model gives a slightly better fitting based on the lower RMSE value compared to the first model as expected. At this point, it is noted that the estimation accuracy of both models could be potentially improved if more data were available. In addition, depending on the requirements of the intended application, the results suggest that the accuracy of the system can be slightly improved with the inclusion from the possible contribution of temperature and relative humidity parameters.

A close examination of Figure 10 reveals that the estimates of PM2.5 concentrations by the linear regression models are shifted with respect to the output of the certified optical particle sizer. Thus, the cross-correlations of the corresponding time series were calculated in order to determine the time delay between them. After determining the lag, where the time series are best aligned by calculating the argmax of the cross-correlation function, the time delay was eliminated in each case and the error metrics for the mobile run test were recalculated, as shown in Table 2. Specifically, the time series are best aligned at lag 1, which corresponds to a time delay of 1 min given that the time series were averaged to 1 min resolution. The error values of the estimated PM2.5 concentrations by the linear regression models with respect to the readings of TSI OPS 3330 are significantly reduced after the lag correction in both cases. This finding suggests that the response of the low-cost APMS presents a delay when compared to the certified optical particle sizer that affects the estimation accuracy during mobile applications. On the one hand, this delay may be attributed to the properties and limitations of the specific low-cost PM2.5 sensor that was employed. On the other hand, it should be emphasized that the mobile test run was performed with the Mobile Lab under urban traffic conditions, where abrupt changes of PM2.5 concentrations were observed, as also revealed by the spikes in Figure 10, possibly due to the high spatial dispersion of the measurements in an urban environment while the Mobile Lab was in motion and thus the potentially high difference of PM2.5 concentrations between successive measurements. Nevertheless, it is noted that the adjusted output of the low-cost APMS (as derived from the regression models) maintains a sufficient level of accuracy in both cases, that is, with and without lag correction, under the conditions of the mobile test run, where abrupt variations in PM concentrations are likely to occur. It is also clarified that: (i) the results in Table 1 and Table 2 refer to the errors metrics of the output of the linear regression models in the frame of the mobile test run; (ii) the regression models were derived from the measurements of the stationary experiment (described in Section 4); and (iii) the stationary experiment reflects different conditions with potentially smoother changes of PM2.5 concentrations between successive measurements.

## 6. Case Study

**Figure 11:**
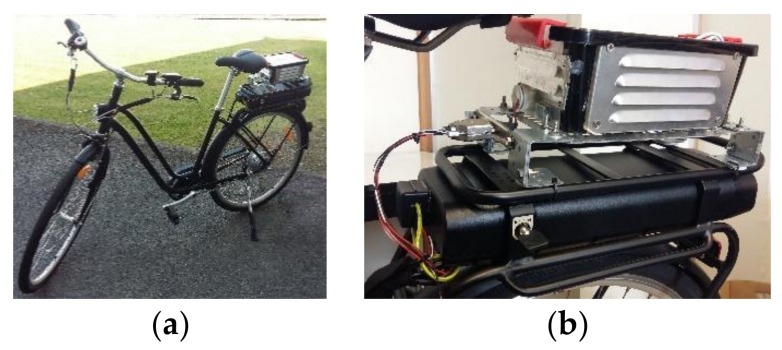
Details of low-cost APMS installation for the intended application: (**a**) Device mounting on e-bike; (**b**) Power supply by the battery of the e-bike.

**Figure 12:**
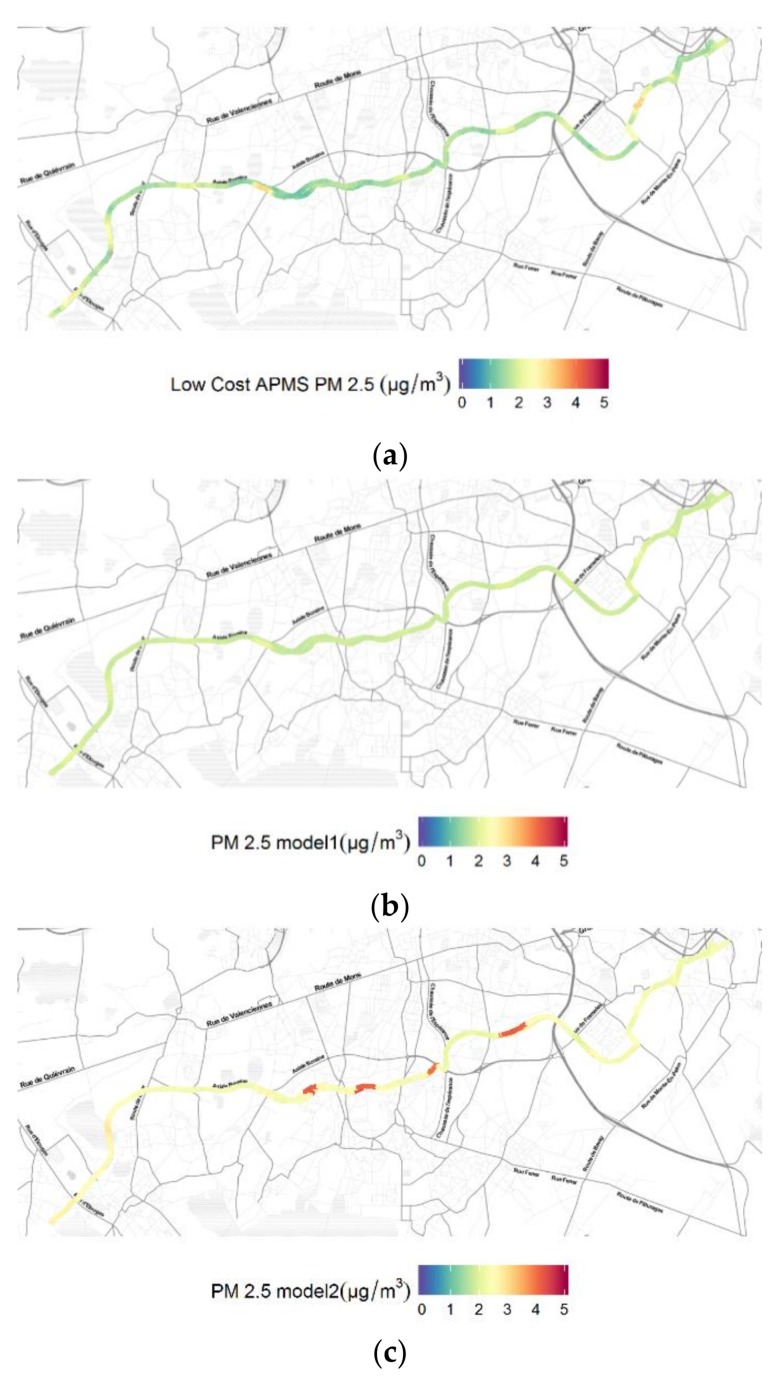
Heat maps of PM2.5 concentrations during the e-bike run at the outskirts of Mons on 6 August 2017: (**a**) Raw measurements of the on-board PM sensor; (**b**) Output of linear regression model 1; (**c**) Output of linear regression model 2.

The low-cost APMS is further equipped with an on-board GPS module, enabling the data logger to capture directly in real-time geo-referenced data that are relevant to the exposure of road users to ambient PM concentration levels. To demonstrate the intended application with respect to the necessity for collecting fine-grained spatio-temporal PM2.5 profiles in an urban setting, the proposed APMS was employed for an e-bike run in Mons, Belgium on 6 August 2017. Specifically, the developed low-cost device was mounted on an e-bike and was powered by the battery of the e-bike, as shown in Figure 11. As a case study, the e-bike followed a route at the outskirts of the city from 19:05 to 20:45 in order to collect measurements of PM2.5 concentrations. Figure 12 illustrates the raw measurements collected by the low-cost PM2.5 sensor under study along the route as well as the corresponding output of the two fitted linear regression models, using heat maps with the same scale. A first observation from the comparison of the results is that model 1 smoothens the extreme values measured by the low-cost PM2.5 sensor, as expected from Equation (1) that scales the raw PM2.5 concentration values by a factor of 0.216347, while model 2 increases the baseline of the PM2.5 concentration values and introduces additional extreme values in the data series compared to the raw measurements, taking into account the contribution of temperature and relative humidity. 

**Figure 13:**
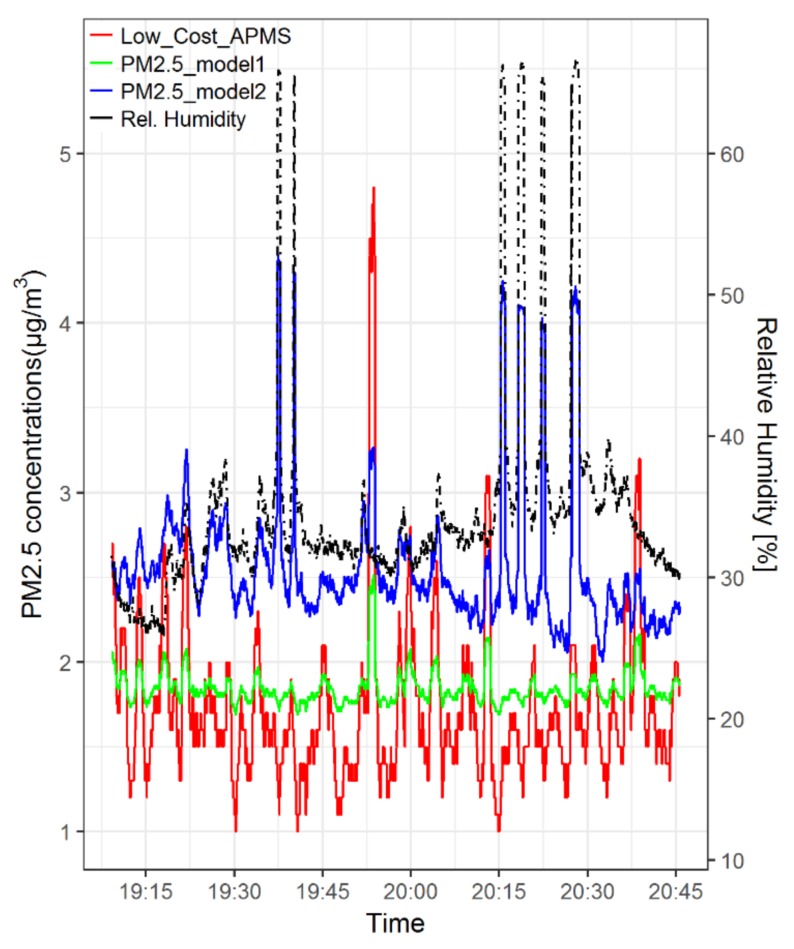
Time series of PM2.5 concentrations with 1 min resolution and relative humidity measurements from the e-bike run in Mons, Belgium on 6 August 2017. The two fitted linear regression models are compared with the raw measurements of the low-cost PM2.5 sensor.

Figure 13 shows the time series of PM2.5 concentration estimates of using the two regression models versus the raw measurements of the low-cost PM2.5 sensor. The comparison of the time series confirms that model 1 produces values within a shorter range, whereas model 2 produces higher estimates at points where the low-cost PM2.5 sensor did not reach peak values. This can be attributed to the linear dependency of model 2 on relative humidity as denoted in Equation (2), given that a careful examination of Figure 13 clearly indicates that the spikes of PM2.5 concentrations produced by model 2 coincide with the spikes of relative humidity measurements. At this point, it is important to emphasize that the observed spikes of relative humidity are due to the fact that the e-bike was passing by dense vegetation consisting of tall trees and thus do not necessarily constitute outliers. It is noted, however, that both the raw measurements and the estimates of the regression models are within the range from 1 to 5 µg/m^3^.

For the sake of completeness, the results of the e-bike run were compared to the values of PM2.5 concentrations retrieved from the Belgian Interregional Environment Agency portal [32]. The installed instrument for measuring PM2.5 concentrations at the monitoring station of Mons is a GRIMM Portable Laser Aerosol spectrometer and Dust Monitor Model 1.108/1.109. Readings from this station are available with an hourly resolution and for the specific time frame on 6 August 2017 the average values recorded were 6.5 μg/m^3^ at 19:00 h, 5.5 μg/m^3^ at 20:00 h and 5 μg/m^3^ at 21:00 h. Despite the accuracy of the expensive equipment installed at the fixed monitoring station (compared to the cost of the APMS proposed in this work), the hourly average values reflect a coarse-grained estimate of the exposure of the general population in the area to PM2.5 concentrations, taking into account that the station is located 6.3 km away from the beginning of the test route followed by the e-bike. Combining all the above, the low-cost APMS appears to underestimate the PM2.5 concentrations compared to the measurements by the fixed monitoring station, even with the model 2 that increases the baseline of the raw measurements, yet it provides a compact, portable and inexpensive solution for capturing in real-time geo-referenced data of PM2.5 concentrations in order to create fine-grained spatio-temporal profiles.

## 7. Conclusions

This paper examines whether the proposed low-cost implementation can provide a promising alternative for an integrated APMS dedicated to PM2.5 concentration estimation with sufficient accuracy. To that end, the calibration of the developed system was performed with a Mobile Lab equipped with a commercially available optical particle sizer as a certified comparison instrument in a roadside stationary test. Then, the detection of this category of contaminants was tested in a mobile run under an urban traffic scenario in order to validate the ability of the proposed system to capture fine-grained spatio-temporal data of environmental interest. The observed delay between the measurements of the comparison instrument and the low-cost APMS may be attributed to the properties and limitations of the specific low-cost PM2.5 sensor that was employed. Despite of the errors introduced due to the conditions of the mobile test run, the derived regression models (which were developed to adjust the output of the low-cost APMS) maintain a high level of accuracy. Hence, depending on the requirements of the intended application, this lack of very fast response could be overcome by replacing the current component with a PM sensor that is more responsive to abrupt changes. Last, this paper presented a case study to highlight the necessity of collecting high-resolution geolocation data of PM2.5 concentrations in an urban setting by comparing the measurements collected by the low-cost APMS mounted on an e-bike with those retrieved from the local fixed monitoring station in Mons.

The accuracy of the system can be improved with further on-field calibration and validation. At first, a wider range of PM2.5 concentrations must be accessed in order to reduce the residual errors observed when measuring higher concentrations. More specifically, the possibility to examine a data set from a longer run can result in a more precise data analysis, where the model coefficients can be derived using cross-validation in order to determine the best values for the coefficients of the fitted models. At this point, it is important to note that the World Health Organization (WHO) guidelines set the limit for the annual mean of PM2.5 to 10 μg/m^3^ [33]. In this regard, the proposed low-cost APMS is fully capable of capturing incidents where this threshold value is exceeded. Furthermore, a limitation of the proposed implementation is the risk of accumulating dust inside the PM sensors’ optical chamber, thus affecting the accuracy of the measurements. To this end, careful cleaning of the chamber must be executed occasionally as part of the systems’ maintenance routine. Thus, future work could focus on the study of dust accumulation effects on the accuracy of the sensor. Last, other directions of future work include a comparative analysis with at least one other commercially available low-cost PM sensor and already characterized in other publications, as well as a reproducibility study based on more low-cost PM sensors of the same type.

## Figures and Tables

**Table 1:** Error metrics for the mobile run test (without lag correction).

Model	RMSE (μg/m^3^)	MAE (μg/m^3^)	MAPE (%)
PM2.5_model1_	2.26	1.76	22.3
PM2.5_model2_	1.97	1.56	21.6

**Table 2:** Error metrics for the mobile run test (with lag correction).

Model	RMSE (μg/m^3^)	MAE (μg/m^3^)	MAPE (%)
PM2.5_model1_	1.80	1.40	17.21
PM2.5_model2_	1.40	1.11	15.21
